# Intragenerational social mobility and cause-specific premature mortality

**DOI:** 10.1371/journal.pone.0211977

**Published:** 2019-02-08

**Authors:** Sunnee Billingsley

**Affiliations:** Department of Sociology, Stockholm University, Stockholm, Sweden; University of Jyvaskyla, FINLAND

## Abstract

This study explores whether there is a short-term relationship between intragenerational social mobility and mortality while individuals are working and whether it is widespread across different causes of death. Net of accumulated advantages and disadvantages, social mobility may influence mortality through health selection or changes in well-being. Men and women working in 1996 up to age 65 are observed annually until 2012 in Swedish register data. Time-varying covariates and origin and destination status are controlled for in discrete time event-history analyses. Results show that when men were upwardly mobile, mortality was lower due to cancer, CVD, IHD, and suicide. Upward mobility was only associated with lower odds of suicide for women. When downwardly mobile, cancer mortality was higher for both men and women and smoking-related cancer mortality was higher for men. Social mobility was not linked to deaths related to accidents and poisoning or alcohol-related mortality. The results may support a relationship between social mobility and mortality characterized by health selection: Only in the case of a chronic illness (cancer) was downward mobility associated with higher mortality. The widespread relationship between upward mobility and lower mortality for men may also indicate positive health selection into attaining a higher class and that individuals with poor health may be less likely to search for better positions or receive promotions.

## Introduction

Developments in life course research have increasingly supported the idea that mortality risk is influenced by socioeconomic position in a cumulative process [1–3]. Upwardly mobile individuals have better mortality rates than those in their origin class but worse health than those in their destination class, and the reverse relationship appears for downwardly mobile individuals [4,5]. These findings are interpreted as evidence for two mechanisms that link mobility and mortality. First, they may indicate social selection [6]: poor health or health behavior leads to downward mobility, whereas good health promotes upward mobility. Second, these findings may reflect social causation: individuals are exposed to health risks and benefits associated with the culture and environment of both classes [2,7].

This literature has primarily focused on the contribution of mobility to inequalities in mortality across social classes [1,4,14–16,5,6,8–13] and the standard research design has been to compare social class at widely spaced moments in time. In Sweden, as in the international literature, researchers studying intragenerational mobility and mortality used mobility measures based on adults’ occupations with generally a 10–25 year gap in between measures [16–19]. But if poor health is a strong enough factor to cause downward mobility, it may also be that mortality risks are affected in the immediate years. Due to the long time span between measures, the possibility of a short-term association between mobility and mortality has not yet been observed in research: individuals may have died before the second class measure or they may have exited the labor market after mobility. Moreover, by not following career trajectories after the second measure of class, later mobility events are unobserved. This study explicitly aims to capture short-term relationships between mobility and mortality by continual observation of class and mortality, which allows the possibility of direct selection as well as other mechanisms.

In addition, the relationship between mortality and mobility is estimated net of accumulated advantage and disadvantage in this study: Social causation arguments involve the combined influence of origin and destinations status, which in the broader social mobility research has been called the “null hypothesis” [20] because it supposes only an additive influence of past and current social status and that mobility has no independent relationship to the outcome. By accounting for these additive effects and focusing on mobility independently, we assume the relationship may be driven by other factors than past and current work conditions (including occupational hazards), prestige or resources such as income. Changes in an individual’s employment situation may influence mortality through changes in well-being, stress in particular. Stress and/or stress-related psychiatric disorders are linked to increased risk of cardiovascular disease [21,22] and suicide [23]. By threatening individuals’ perceived social standing, downward mobility in particular may influence health by inhibiting self-regulation and leading to poor health behavior [24].

Selection and causal pathways are not disentangled in this study, rather the aim is to observe the relationship when the research design more fully allows selectivity into mobility to be operative. The second contribution of the study is to explore whether the relationship between mobility and mortality is widespread across different causes of death. Cause-specific mortality is distinguished along the lines of physical disease and disorders including deaths due to physical illness such as cardiovascular diseases, cancer, ischemic heart disease, and stroke; health behavior, including deaths due to alcohol consumption and smoking; psychiatric or emotional health including suicide; and external causes of deaths, including accidents and poisoning. Health selection may be more linked to chronic causes of death because longer-term illnesses are more likely to influence careers before death. We may therefore expect, for example, that deaths related to cancer and health behavior are linked to downward mobility, but not necessarily accidents. Achieving career goals has been linked to not smoking and low alcohol consumption [25], so we may also expect upward mobility to be linked to lower mortality related to health behavior.

In contexts with well-developed job protection policies and social benefits such as Sweden [26], individuals do not easily lose a job due to poor health. However, individuals suffering from limited health may choose to step down in their careers to lessen demands and responsibilities. Another contextual factor to consider is the highly gender segregated nature of the Swedish labor market. Men and women should be studied separately to account for differential processes into mobility related to this segmentation and because mortality in general has been more strongly linked to career developments for men than women [27].

## Materials and methods

Data used for the study come from multiple harmonized population-based registers provided by Statistics Sweden, including annual information on basic demographic information, education, occupation and mortality from 1996 until 2012. The sample includes men and women of working age (18–65) with an occupation recorded in 1996. We observe 1137436 women and 825140 men; we have fewer men because they are over-represented in small private firms, which are not completely covered in our data as described below.

### Dependent variable

We distinguish all-cause mortality and cause-specific mortality from the Swedish cause of death register, which uses ICD-10 over the period 1996–2012: all cancers (C00-C97, D00-D48); smoking-related cancer (C00-C06, C09-C15, C25, C30-C34, C38, C64-C68); breast cancer (C50); prostate cancer (C61); cardiovascular diseases (CVD) (I00-199); ischemic heart disease (IHD) (I20-I25); stroke/cerebrovascular diseases (I60-I69); alcohol-related causes combined (E24.4, F10, G31.2, G62.1, I42.6, K29.2, K70, K85.2, K86.0, O35.4, P04.3 Q86, T51, X45, Y90.1-Y90.9, Y91.1-Y91.9, Z50.2, Z71.4, Z72.1 [28]); suicide including undetermined causes of death (X60-X84, Y10-Y34); transport accidents, accidental fall and poisoning (V01-V99, W00-W19, X40-X49). The mortality indicator is adjusted so that mortality in a given year is aligned with occupational information from the previous year.

### Independent variables

The occupational register includes all individuals working in public firms, and all individuals working in private firms with > = 500 employees. Smaller private firms are randomly sampled on the basis of size, where employees of larger firms have a higher chance of being included. We imputed the previous occupation when it was missing for a certain year due to a firm not being sampled and income remained similar (<10% difference). All occupational information is collected in the fall (October or November) and reflects employment in that month. The social class measure derives from Swedish occupational codes, which are decoded into three digit ISCO88 codes, and translated into the European Socioeconomic Classification (EseC). Because the occupational register does not include information on the self-employed and small employers, we only have seven possible classes, which are collapsed into four in order to achieve a more hierarchical schema. The class categories are grouped accordingly: 1) EseC 1 (high): large employers; professional, administrative and managerial occupations; higher grade technician and supervisory occupations; 2) EseC 2 (intermediate): intermediate occupations and lower supervisory or lower technician occupations, 3) EseC 3 (white collar workers): lower services, sales and clerical occupations; 4) EseC 4 (blue collar workers): lower technical occupations and routine occupations.

Origin class is defined as the occupational class in the first observed year (1996) and is held constant over all following years. As is usual, the timing of this measure reflects the first measure available in the data. Destination class is the occupational class in each year thereafter, or indicates whether an individual was not employed (had no labor income because of being unemployed, retired, sick leave or disability the entire year), studying, or missing information. Missing information means that the individual did have labor earnings that year, but no accompanying occupational information, which could be due to self-employment, unemployment at the moment occupational information was registered, or working in a smaller private firm that was not sampled. The social mobility variable is coded according to the following: 1) downward mobility: destination class is lower (higher EseC designation) than the origin class; 2) upward mobility: destination class is higher (lower EseC designation) than origin class; 3) no mobility: destination and origin class are the same. The mobility measure also includes a missing category that overlaps with the destination categories of not employed, studying or missing information (when two independent variables share an overlapping category, one of the variable’s overlapping category is automatically omitted from the model). Individuals who retire before being censored at age 66 are classified as not employed and therefore not at risk of social mobility (not applicable/missing), as in the case of studying, not employed for other reasons or missing information. Because social mobility is not possible in the year we identify the origin status, the analyses cover the years 1997–2012.

All additional control variables are time-varying and include: 1) resides in a metropolitan area (Stockholm, Malmö or Gothenburg), or small city/rural area; 2) married/registered partnership, unmarried, or prior marriage/registered partnership; 3) enrolled in education, or finished education with i) < = 2 years of secondary education, ii) >2 years of secondary education, iii) < = 3 years higher education, or iv) >3 years higher education; 4) born in Sweden or foreign born.

### Method

We used discrete time event-history analysis, which is useful for binary outcomes and covariates that are measured on an annual basis [29]. The specification is as follows

$$\log\left(\frac{P_{it}}{1-P_{it}}\right)=\alpha D_{it}+\beta C_{it}+\beta V_{it}$$

where *P_it_* is the probability of death during interval *t*. *D_it_* is a vector of dummies for age groups defined in five-year intervals, which serve as the duration with coefficients *α*. *C_it_* serves as a vector of covariates that are constant over time—origin class and country of birth—with coefficients *β*. *V_it_* is a vector of covariates that vary over time, including educational level, residential location, partnership status, social mobility and destination class. Individuals are censored when they emigrated, died or at the end of the study (in 2012).

The Diagonal Reference Model (DRM) [30,31] best separates the effect of social mobility from the effects related to origin and destination class according to social stratification and mobility research. Previous research on this data [32] compared results from a DRM and the approach used in the current study and no substantial difference between estimates were found. This implies that controlling for origin and destination status sufficiently accounts for accumulated advantages and disadvantages in this research setting, as well as floor and ceiling effects related to no possibility for workers in the lowest class to be downwardly mobile and workers in the highest class to be upwardly mobile. The DRM was not used because of complications incorporating destination statuses that are not symmetrical to origin status (ibid).

## Results

The characteristics of the study sample (“population” in the case of public employees and private employees in large firms) are presented in S1 Table. Table 1 shows the cause of death distribution for men and women (the percentages do not sum to 100% because individuals may contribute to more than one category). For working-aged adults who were employed in 1996, overall cancer and CVD deaths are most common among both men and women, while causes of death related to accidents and poisoning and alcohol consumption are least common for both sexes.

**Table 1:** Cause of death distribution across sample, % of all deaths for men and women separately.

	Men	Women
Total deaths observed	25,352	24,773
All cancer	38.3	63.9
Prostate cancer	3.1	-
Breast cancer	-	13.1
CVD	29.9	14.7
IHD	18.5	6.3
Stroke	4.1	4.5
Smoking-related cancer	14.3	18.7
Alcohol-related mortality	3.8	1.8
Accidents/poisoning	4.3	2
Suicide	7.6	4.4

Tables 2 and 3 display, respectively, the share of men’s and women’s person-years spent in each origin, destination and social mobility status and the share of these observations that ended in a death for men and women in each of these statuses. Looking first at the change from origin to destination class over the observation period, the overall share of employment exits for blue-collar workers is the greatest (18.8 vs. 7.4 percentage point loss for the highest class) for men, whereas the loss is greatest for women in the white collar working class. This demonstrates differential selection into remaining in employment for various classes. Mortality is highest in the category of “no activity” in the destination class variable (which heavily overlaps with “not applicable/missing” in the social mobility variable) because individuals with the poorest health leave the labor market. Among men, we see high mortality due to all-cause cancer and CVD in the “no activity” group, while less so but still high mortality due to IHD and smoking-related cancer in this group of not employed men. Among women who die from CVD, fewer exit the labor market beforehand. Before adjusting for age and other important covariates, we often observe lower mortality when individuals have been upwardly mobile, particularly for men, but only higher mortality for all-cause mortality or deaths due to cancer when men and women have been downwardly mobile.

**Table 2:** Percent of all person-years for men in each status and percent of person-years ending in death by origin-, destination social class and social mobility.

Men	% person- years	All mortality	All cancer	Prostate cancer	CVD	IHD	Stroke	Smoking-related cancer	Alcohol-related mortality	Accidents/ poisoning	Suicide
*Origin social class*											
High	37.00	0.20	0.09	0.01	0.06	0.04	0.01	0.03	0.01	0.01	0.01
Intermediate	11.2	0.21	0.09	0.01	0.06	0.04	0.01	0.03	0.01	0.01	0.01
White collar workers	9.7	0.19	0.07	0.00	0.05	0.03	0.01	0.03	0.01	0.01	0.02
Blue collar workers	42.1	0.25	0.09	0.01	0.08	0.05	0.01	0.03	0.01	0.01	0.02
*Destination class*											
High	29.6	0.12	0.05	0.00	0.04	0.02	0.01	0.02	0.00	0.01	0.01
Intermediate	6.9	0.13	0.05	0.00	0.04	0.03	0.01	0.02	0.00	0.01	0.01
White collar workers	5.5	0.13	0.04	0.00	0.05	0.03	0.01	0.02	0.00	0.01	0.02
Blue collar workers	23.3	0.16	0.05	0.00	0.06	0.04	0.01	0.02	0.01	0.01	0.02
Studying	1.7	0.10	0.02	0.00	0.02	0.02	0.00	0.01	0.01	0.01	0.01
No activity	5.3	0.88	0.27	0.03	0.27	0.16	0.04	0.11	0.06	0.03	0.05
Missing	27.7	0.22	0.13	0.01	0.08	0.05	0.01	0.05	0.01	0.01	0.02
*Social mobility*											
Downward	3.2	0.17	0.07	0.00	0.05	0.03	0.01	0.03	0.00	0.01	0.01
No mobility	54.6	0.14	0.05	0.00	0.05	0.03	0.01	0.02	0.00	0.01	0.01
Upward	7.5	0.09	0.03	0.00	0.03	0.02	0.00	0.01	0.00	0.01	0.01
Not applicable/missing	34.7	0.38	0.15	0.01	0.11	0.06	0.01	0.06	0.02	0.01	0.02

Note: percentages are rounded and 0.00 indicates a percentage less than 0.00 percent of all person years (not 0 deaths)

**Table 3:** Percent of all person-years for women in each status and percent of person-years ending in death by origin-, destination social class and social mobility.

Women	% person- years	All mortality	All cancer	Prostate cancer	CVD	IHD	Stroke	Smoking-related cancer	Alcohol-related mortality	Accidents/ poisoning	Suicide
*Origin social class*											
High	29.00	0.14	0.10	0.02	0.02	0.01	0.01	0.02	0.00	0.00	0.01
Intermediate	20.2	0.16	0.11	0.02	0.02	0.01	0.01	0.03	0.00	0.00	0.01
White collar workers	35.7	0.15	0.09	0.02	0.02	0.01	0.01	0.03	0.00	0.00	0.01
Blue collar workers	15.1	0.20	0.11	0.02	0.04	0.02	0.01	0.04	0.00	0.00	0.01
*Destination class*											
High	24.3	0.08	0.05	0.01	0.01	0.00	0.00	0.01	0.00	0.00	0.00
Intermediate	12.9	0.09	0.06	0.01	0.01	0.01	0.01	0.02	0.00	0.00	0.00
White collar workers	19.9	0.08	0.05	0.01	0.02	0.01	0.01	0.02	0.00	0.00	0.01
Blue collar workers	7.6	0.11	0.06	0.01	0.03	0.01	0.01	0.02	0.00	0.00	0.01
Studying	3.2	0.05	0.02	0.01	0.01	0.00	0.00	0.01	0.00	0.00	0.01
No activity	5.7	0.26	0.29	0.06	0.10	0.05	0.03	0.09	0.02	0.01	0.02
Missing	26.4	0.16	0.19	0.04	0.03	0.01	0.01	0.05	0.00	0.00	0.01
*Social mobility*											
Downward	2.7	0.10	0.07	0.01	0.02	0.01	0.01	0.02	0.00	0.00	0.00
No mobility	55.1	0.09	0.05	0.01	0.02	0.01	0.01	0.02	0.00	0.00	0.00
Upward	7.00	0.06	0.04	0.01	0.01	0.00	0.00	0.01	0.00	0.00	0.00
Not applicable/missing	35.2	0.29	0.19	0.04	0.04	0.02	0.01	0.05	0.01	0.00	0.01

Note: percentages are rounded and 0.00 indicates a percentage less than 0.00 percent of all person years (not 0 deaths)

Table 4 shows selected model results from discrete time hazard analyses of death due to all causes (full results available in S2 Table). In the first model, when adjusting for control variables and origin class only, the traditional gradient appears for origin class for men in which mortality is lowest for the highest class (OR 0.89, 95% CI 0.85–0.93), but mortality is only elevated for women who worked in the lowest origin class (OR 1.20, 95% CI 1.15–1.25). When origin status is excluded and the current status in each year is included instead, we see a somewhat different pattern. For men, the highest class is still associated with a lower odds of mortality, but the elevated risk in the lowest two classes disappears. The heightened risk is instead observed in the categories of no activity (OR 3.16, 95% CI 2.96–3.38), when individuals had left the labor market, and missing (OR 1.77, 95% CI 1.66–1.88), which can include those who left the labor market as well as those who have no occupational information registered because they are self-employed, unemployed or their firm was not sampled, but had labor earnings that year. Women’s gradient adjusted as well when observing current class and labor market status; the odds ratio for white collar workers declined to 0.89 (95% CI 0.84–0.95) and inactive women had an odds ratio of 4.03 (95% CI 3.81–4.26) and those with missing information had an odds ratio of 2.58 (95% CI 2.45–2.71). Because these relationships are based on individuals’ class and labor market status a short time before death, a selection interpretation of these altered patterns would indicate that men in the lowest class and women in white collar occupations do not continue working when ill, whereas men and women in other classes maintain a stronger connection to the labor market.

**Table 4:** Odds ratios (and 95% confidence intervals) from discrete time hazard analysis of all-cause mortality: Origin class, destination class and mobility, 1997 to 2012, adjusted for control variables.

	Men			Women		
* *	Origin status model	Destination status model	Origin, destination and social mobility	Origin status model	Destination status model	Origin, destination and social mobility
*Origin social class*						
High	0.89 (0.85–0.93)		0.96 (0.91–1.01)	0.99 (0.94–1.03)		1.11 (1.06–1.16)
Intermediate	1		1	1		1
White collar workers	1.07 (1.01–1.14)		1.10 (1.03–1.17)	0.98 (0.94–1.01)		0.96 (0.92–1.00)
Blue collar workers	1.10 (1.05–1.15)		1.15 (1.10–1.21)	1.20 (1.15–1.25)		1.07 (1.02–1.13)
*Destination class*						
High		0.91 (0.85–0.98)	0.94 (0.87–1.01)		0.98 (0.92–1.04)	0.94 (0.88–1.01)
Intermediate		1	1		1	1
White collar workers		0.96 (0.87–1.05)	0.92 (0.83–1.02)		0.89 (0.84–0.95)	0.93 (0.86–0.99)
Blue collar workers		1.00 (0.93–1.07)	0.89 (0.83–0.96)		1.14 (1.06–1.23)	1.07 (0.99–1.16)
Studying		omitted	omitted		omitted	omitted
No activity		3.16 (2.96–3.38)	2.95 (2.74–3.18)		4.03 (3.81–4.26)	3.99 (3.75–4.24)
Missing		1.77 (1.66–1.88)	1.66 (1.54–1.78)		2.58 (2.45–2.71)	2.57 (2.43–2.72)
*Social mobility*						
Upward			0.78 (0.72–0.84)			1.07 (0.97–1.18)
No mobility			1			1
Downward			1.06 (0.97–1.15)			0.99 (0.91–1.07)
Number of observations	11410734	11410734	11410734	15769310	15769310	15769310
Log likelihood	-169285.9	-167257.5	-167208.5	-175829.5	-172478.6	-172449.6
AIC	338613.8	334561	334473	351701	345003.1	344955.2
BIC	338913.1	334888.8	334872	352007	345338.3	345363.3

Note: results additionally adjusted for age, marital status, foreign born, residence and education. Full model results for the final model (including origin class, destination class and social mobility) are provided in S1 Table.

The final column for men and women reveals the combined effect of accumulated advantage and disadvantage (origin and destination status) and social mobility. Similar patterns emerge, but with some evidence that variance in mortality becomes partitioned differently between origin and destination status for a few classes. Despite the descriptive statistics, downward social mobility does not appear linked to heightened mortality overall for either men or women. In contrast, upward mobility is related to a 22% lower odds of death for men (OR 0.78, CI 0.72–0.84).

Table 5 displays selected results of cause-specific mortality analyses. S1 and S2 Figs plot the same results to give a visual representation of the differences across causes of death. The relationship between cancer-related mortality and social mobility operates very differently for men and women. Net of selectivity out of specific classes, the odds of death due to cancer decline when upwardly mobile for men (OR 0.79, 95% CI 0.69–0.90) and when downwardly mobile the odds of death increase for both men (OR 1.22 95% CI 1.06–1.40) and women (OR 1.14 95% CI 1.00–1.29). The odds of death are also increased (although the confidence interval overlaps with 1) for women who are upwardly mobile. Premature cancer mortality in women is mostly driven by breast cancer mortality, which does not follow a traditional negative class gradient. No clear pattern emerges for breast cancer and social mobility, however. Men, on the other hand, had lower odds of prostate cancer-related death when upwardly mobile (OR 0.47 95% CI 0.24–0.94).

**Table 5:** Selected model results for cause-specific mortality from 1997 to 2012: Odds ratios (and 95% confidence intervals) from discrete time hazard analysis results.

	Men	Women
***Cancer***		
Upward	0.79 (0.69–0.90)	1.09 (0.98–1.21)
No social mobility	1	1
Downward	1.22 (1.06–1.40)	1.14 (1.00–1.29)
***Breast cancer***		
Upward		1.11 (0.89–1.39)
No social mobility		1
Downward		1.12 (0.83–1.50)
***Prostate cancer***		
Upward	0.47 (0.24–0.94)	
No social mobility	1	
Downward	1.34 (0.74–2.41)	
***CVD***		
Upward	0.74 (0.64–0.86)	0.85 (0.68–1.05)
No social mobility	1	1
Downward	0.98 (0.84–1.15)	1.04 (0.82–1.33)
***IHD***		
Upward	0.73 (0.60–0.88)	0.89 (0.63–1.26)
No social mobility	1	1
Downward	0.99 (0.82–1.21)	0.94 (0.64–1.40)
***Stroke***		
Upward	0.72 (0.48–1.09)	0.86 (0.59–1.24)
No social mobility	1	1
Downward	1.04 (0.68–1.57)	1.08 (0.74–1.59)
***Smoking-related cancer***		
Upward	0.63 (0.50–0.80)	1.05 (0.86–1.27)
No social mobility	1	1
Downward	1.32 (1.05–1.65)	1.21 (0.97–1.51)
***Alcohol related mortality***		
Upward	0.57 (0.32–1.01)	0.69 (0.29–1.64)
No social mobility	1	1
Downward	0.59 (0.32–1.10)	0.75 (0.27–2.09)
***Suicide***		
Upward	0.76 (0.59–0.98)	0.65 (0.44–0.97)
No social mobility	1	1
Downward	0.74 (0.52–1.05)	0.95 (0.60–1.50)
***Accidents/poisoning***		
Upward	0.74 (0.52–1.04)	1.00 (0.62–1.64)
No social mobility	1	1
Downward	0.78 (0.49–1.24)	0.93 (0.49–1.76)

Note: results adjusted for age, education, foreign origin, marital status, residence, origin class and destination class

CVD and IHD related deaths showed very similar relationships to upward mobility for men, where the odds of death were reduced by over 25%. No consistent relationship existed for downward mobility in relation to these illnesses for men and no relationships between mobility in either direction and stroke-related mortality appeared. Women showed no relationship between social mobility and death due to CVD, IHD or stroke.

Estimates for health-behavior related mortality reveal that men have lower mortality due to smoking-related cancer when upwardly mobile compared to when not mobile (OR 0.63, 95% CI 0.50–0.80) and a higher odds of mortality when downwardly mobile (OR 1.32 95% CI 1.05–1.65). Women’s smoking-related mortality was not linked to social mobility and no relationship between mobility and alcohol-related mortality appeared for either men or women.

Suicide was inversely associated with upward mobility for both men and women, respectively (OR 0.76, 95% CI 0.59–0.98; OR 0.65, 95% CI 0.44–0.97). Social mobility was not linked to deaths related to accidents and poisoning.

## Discussion

This study revealed previously unobserved aspects of the relationship between intragenerational social mobility and mortality by allowing mobility and mortality to be linked close in time and accounting for the continual possibility of mobility and class exits such as those due to poor health. Although individuals may be observed for as long as 16 years (from 1996–2012), the relationship between mobility and mortality estimated here uniquely reflects short-term relationships as well as direct selection effects because of continued annual observation. Relationships operating over this period may, however, also reflect other mechanisms such as changes in stress or well-being.

A predominant finding of this study is that men’s mortality is inversely linked to upward mobility in Sweden but rarely linked to downward mobility. Men are less likely to die when they have been upwardly mobile from a variety of causes of death including physical diseases, health related behavior, and suicide. Without disaggregating causes of death, we find that downward social mobility is not related to mortality for either men or women and only upward mobility is related to mortality for men. That mobility is not associated with all-cause mortality among women means that overall variation in women’s mortality can primarily be explained by cumulative exposure to class advantages and disadvantages. These findings contrast those with similar data and research design [32] in which mortality was heightened for both men and women when they had been downwardly mobile. The difference between the findings is due to the earlier study not observing destination class after 2007, which demonstrates the unique relationship observed if up to date destination status is modeled constantly.

Regardless of different modeling approaches, little research exists on cause-specific mortality and social mobility to which the results here can be compared. Unlike Hart et al. [1], who found no relationship between social mobility and cancer mortality in Scotland, we find decreased odds of cancer mortality (including prostate and smoking-related cancer) among upwardly mobile men and increased odds for both men and women who were downwardly mobile. We found no relationship between mobility and breast cancer mortality, similar to studies on breast cancer incidence and women’s occupational mobility [33] or occupational class [34].

Upward mobility appears to be associated with lower odds of suicide among men and women. When considering death due to health-related risk behavior, upward mobility was related to lower odds of smoking-related cancer death (among men). Mobility was not linked to alcohol-related causes of mortality, which likely relates to alcohol-related mortality occurring mostly outside the workforce because high alcohol consumption can reduce labor force attachment years before death [35]. This may be why no relationship appeared in past research that combined smoking and alcohol-related cancers [34].

The increased risk of mortality (all or cause specific) from downward mobility found in other studies (10–12, 13, 30, 34) but rarely in this study, might be related to the analytical differences such as our short-term focus and separating the effect of cumulative class exposure from that related to mobility. But certain financial or policy settings may also play a role; in contexts of severe economic crisis and uncertainty such as Russia after the fall of the Soviet Union [36] and in Albania after a nationwide pyramid savings scheme collapsed [37], stress has been identified as a leading mechanism linked to downward mobility. However, the role of context is not clear, as other findings [38] show that economic growth was the period during which the relationship between downward mobility and mortality was strongest in Finland.

The results of our study provide only weak support at best for theories of causal effects related to stress because downward mobility was related to higher mortality only for cancer. Health selection into mobility is most likely for chronic diseases because the interval between timing of illness onset and mortality may be lengthy enough for downward mobility to occur. This scenario depends on how Swedish workers navigate employment while having poor health. Apart from going on sickness-related full-time leave of absence, they can: 1) work while being ill (perhaps combined with a partial sickness leave), 2) exit the labour market through unemployment or disability pension. Our results suggest that both pathways exist for this population in the immediate years before death. Among men and women who die from cancer, for example, labour attachment appears to be maintained the least for individuals working in the lowest classes. Fighting cancer is likely to be more difficult in physically demanding jobs. In contrast, labour force attachment is maintained in lower class positions preceding alcohol-related mortality or suicide. Higher occupational classes may require higher cognitive and social functioning that would be compromised by high alcohol consumption or psychiatric disorders.

That upward mobility was widely associated with lower mortality for men may partly originate from childhood living conditions and the better health this generates as well as better mobility prospects [39]. From a short-term perspective, however, selection into upward mobility likely also results from ill individuals being less likely to search for higher positions and/or successfully achieve them, as well as being less promoted due to stigma related to their health [40]. Certain policies may therefore support upward mobility prospects for those who have suffered a recent health setback. For example, policies aimed to protect worker’s privacy and support career development after career interruptions such as re-training or skill upgrading over the life course may improve all individuals’ chances of upward mobility. More research is necessary to identify the factors involved in this relationship, however.

A limitation of our data is the missing data on occupations that is not randomly distributed. Self-employed, the temporary non-employed and those working in small private firms are either not represented or underrepresented, and career-driven individuals and men are more likely to be employed in the private sector. Our results are therefore not generalizable to the entire working population of Sweden. Nevertheless, our findings that reflect short-term relationships between intragenerational mobility and specific causes of death offer insight into how health inequalities are created and reproduced.

### Ethical statement

Statistics Sweden de-identifies personal information as well as ensures anonymity in its registers by blocking access to information that would allow identification of an individual.

## Supporting information

S1 Fig Selected results for men’s cause-specific mortality, 1997–2012: Odds ratios (and 95% confidence intervals) from discrete time hazard analysis results, adjusted for age, education, foreign origin, marital status, residence, origin class and destination class. (DOCX)

S2 Fig Selected results for women’s cause-specific mortality, 1997–2012: Odds ratios (and 95% confidence intervals) from discrete time hazard analysis results, adjusted for age, education, foreign origin, marital status, residence, origin class and destination class. (DOCX)

S1 Table Sample description. (DOCX)

S2 Table Full model results of all-cause mortality, 1997–2012. (DOCX)
