# Supplementary material for: Intragenerational social mobility and cause-specific premature mortality
Source: PLoS One. 2019 Feb 8;14(2):e0211977. doi: 10.1371/journal.pone.0211977 (PMC6368327; doi:10.1371/journal.pone.0211977)
Supplement: S2 Fig — (DOCX) [file pone.0211977.s002.docx]

**S2 Figure**. Selected results for women’s cause-specific mortality, 1997-2012: Odds ratios (and 95% confidence intervals) from discrete time hazard analysis results, adjusted for age, education, foreign origin, marital status, residence, origin class and destination class
