# Supplementary material for: Intragenerational social mobility and cause-specific premature mortality
Source: PLoS One. 2019 Feb 8;14(2):e0211977. doi: 10.1371/journal.pone.0211977 (PMC6368327; doi:10.1371/journal.pone.0211977)
Supplement: S1 Table — (DOCX) [file pone.0211977.s003.docx]

**S1 Table. Sample description**

|  | Men (N=825,140) | | Women (N=1,137,436) | |
| --- | --- | --- | --- | --- |
|  | N, (men/years) | %, Share of observations (men/years) | N, (women/years) | % Share of observations (women/years) |
| All | 11,410,734 |  | 15,769,327 |  |
| *Age* |  |  |  |  |
| 17-25 | 176,223 | 1.5 | 233,525 | 1.5 |
| 26-30 | 532,391 | 4.7 | 652,359 | 4.1 |
| 31-35 | 1,034,943 | 9.1 | 1,244,173 | 7.9 |
| 36-40 | 1,414,025 | 12.4 | 1,756,157 | 11.1 |
| 41-45 | 1,609,283 | 14.1 | 2,134,457 | 13.5 |
| 46-50 | 1,680,883 | 14.7 | 2,410,083 | 15.3 |
| 51-55 | 1,794,113 | 15.7 | 2,650,176 | 16.8 |
| 56-60 | 1,733,547 | 15.2 | 2,565,267 | 16.3 |
| 61-65 | 1,435,326 | 12.6 | 2,123,130 | 13.5 |
| *Marital status* |  |  |  |  |
| Married/registered partnership | 6,167,801 | 54.1 | 8,848,814 | 56.1 |
| Unmarried | 3,787,684 | 33.2 | 4,085,907 | 25.9 |
| Prior marriage/registered partnership | 1,455,249 | 12.7 | 2,834,589 | 18 |
| Missing | - | - | 17 | 0 |
| *Country of birth* |  |  |  |  |
| Born in Sweden | 10,499,523 | 92 | 14,441,814 | 91.6 |
| Foreign born | 911,211 | 8 | 1,327,513 | 8.4 |
| *Education* |  |  |  |  |
| In education | 187,504 | 1.6 | 495,156 | 3.1 |
| <=2 years of secondary education | 5,253,540 | 46 | 7,458,748 | 47.3 |
| 2-3 years of secondary education | 1,760,434 | 15.4 | 1,830,695 | 11.6 |
| <=3 years higher education | 1,756,155 | 15.4 | 2,646,540 | 16.8 |
| >3 years higher education | 2,440,504 | 21.4 | 3,330,699 | 21.1 |
| Missing | 12,597 | 0.1 | 7,489 | 0.1 |
| *Residence* |  |  |  |  |
| Rural or small city | 7,920,294 | 69.4 | 10,992,461 | 69.7 |
| Large city | 3,490,440 | 30.6 | 4,776,866 | 30.3 |
| *Origin social class* |  |  |  |  |
| High | 4,220,748 | 37 | 4,572,018 | 29 |
| Intermediate | 1,279,392 | 11.2 | 3,191,870 | 20.2 |
| White collar workers | 1,109,707 | 9.7 | 5,622,797 | 35.7 |
| Blue collar workers | 4,800,887 | 42.1 | 2,382,642 | 15.1 |
| *Destination class* |  |  |  |  |
| High | 3,382,710 | 29.6 | 3,834,720 | 24.3 |
| Intermediate | 787,375 | 6.9 | 2,037,727 | 12.9 |
| White collar workers | 627,458 | 5.5 | 3,146,165 | 19.9 |
| Blue collar workers | 2,652,748 | 23.2 | 1,192,908 | 7.6 |
| Studying | 187,504 | 1.6 | 495,156 | 3.2 |
| No activity | 609,72 | 5.3 | 895,133 | 5.7 |
| Missing | 3,163,219 | 27.7 | 4,167,518 | 26.4 |
| *Social mobility* |  |  |  |  |
| No mobility | 6,228,520 | 54.6 | 8,684,643 | 55.1 |
| Downward | 368,182 | 3.2 | 430,718 | 2.8 |
| Upward | 853,589 | 7.5 | 1,096,159 | 6.9 |
| Not applicable/missing | 3,960,443 | 34.7 | 5,557,807 | 35.2 |
