# Supplementary material for: Intragenerational social mobility and cause-specific premature mortality
Source: PLoS One. 2019 Feb 8;14(2):e0211977. doi: 10.1371/journal.pone.0211977 (PMC6368327; doi:10.1371/journal.pone.0211977)
Supplement: S2 Table — (DOCX) [file pone.0211977.s004.docx]

**S2 Table. Full model results of all-cause mortality, 1997-2012**

|  | Men | Women |
| --- | --- | --- |
| *Age* |  |  |
| 25 or younger | 0.35 (0.28-0.45) | 0.17 (0.12-0.25) |
| 26-30 | 0.33 (0.28-0.39) | 0.27 (0.23-0.33) |
| 31-35 | 0.44 (0.40-0.49) | 0.38 (0.33-0.43) |
| 36-40 | 0.65 (0.59-0.71) | 0.59 (0.53-0.64) |
| 41-45 | 1 | 1 |
| 46-50 | 1.70 (1.59-1.82) | 1.73 (1.62-1.85) |
| 51-55 | 2.94 (2.76-3.12) | 2.73 (2.57-2.91) |
| 56-60 | 4.80 (4.53-5.09) | 3.92 (3.70-4.17) |
| 61-65 | 5.97 (5.63-6.33) | 4.18 (3.93-4.44) |
| *Marital status* |  |  |
| Married/registered partnership | 1 | 1 |
| Unmarried | 1.64 (1.59-1.70) | 1.43 (1.38-1.48) |
| Prior marriage/reg. partnership | 1.67 (1.62-1.72) | 1.37 (1.33-1.42) |
| *Country of birth* |  |  |
| Born in Sweden | 1 | 1 |
| Foreign born | 1.07 (1.03-1.12) | 0.90 (0.86-0.94) |
| *Education* |  |  |
| In education | 1.27 (1.08-1.50) | 1.20 (1.04-1.39) |
| <=2 years of secondary education | 1.17 (1.13-1.22) | 1.32 (1.26-1.39) |
| >2 years of secondary education | 1 | 1 |
| <=3 years higher education | 0.94 (0.89-0.98) | 0.95 (0.89-1.00) |
| >3 years higher education | 0.86 (0.82-0.90) | 0.91 (0.85-0.97) |
| *Residence* |  |  |
| Rural or small city | 1 | 1 |
| Large city | 1.07 (1.04-1.10) | 1.10 (1.07-1.13) |
| *Origin social class* |  |  |
| High | 0.96 (0.91-1.01) | 1.11 (1.06-1.16) |
| Intermediate | 1 | 1 |
| White collar workers | 1.10 (1.03-1.17) | 0.96 (0.92-1.00) |
| Blue collar workers | 1.15 (1.10-1.21) | 1.07 (1.02-1.13) |
| *Destination class* |  |  |
| High | 0.94 (0.87-1.01) | 0.94 (0.88-1.01) |
| Intermediate | 1 | 1 |
| White collar workers | 0.92 (0.83-1.02) | 0.93 (0.86-0.99) |
| Blue collar workers | 0.89 (0.83-0.96) | 1.07 (0.99-1.16) |
| Studying | omitted | omitted |
| No activity | 2.95 (2.74-3.18) | 3.99 (3.75-4.24) |
| Missing | 1.66 (1.54-1.78) | 2.57 (2.43-2.72) |
| *Social mobility* |  |  |
| Upward | 0.78 (0.72-0.84) | 1.07 (0.97-1.18) |
| No mobility | 1 | 1 |
| Downward | 1.06 (0.97-1.15) | 0.99 (0.91-1.07) |
| Number of observations | 11410734 | 15769310 |
| Log likelihood | -167208.5 | -172449.6 |
